# A Domain-Specific Terminology for Retinopathy of Prematurity and Its Applications in Clinical Settings

**DOI:** 10.1155/2018/9237319

**Published:** 2018-04-18

**Authors:** Yinsheng Zhang, Guoming Zhang

**Affiliations:** ^1^School of Management Science and E-Business, Zhejiang Gongshang University, Hangzhou, Zhejiang 310018, China; ^2^Contemporary Business and Trade Research Center of Zhejiang Gongshang University, Hangzhou, Zhejiang 310018, China; ^3^Pediatric Retinal Surgery Department, Shenzhen Key Ophthalmic Laboratory, Shenzhen Eye Hospital and The Second Affiliated Hospital of Jinan University, Guangzhou 518040, China

## Abstract

A terminology (or coding system) is a formal set of controlled vocabulary in a specific domain. With a well-defined terminology, each concept in the target domain is assigned with a unique code, which can be identified and processed across different medical systems in an unambiguous way. Though there are lots of well-known biomedical terminologies, there is currently no domain-specific terminology for ROP (retinopathy of prematurity). Based on a collection of historical ROP patients' data in the electronic medical record system, we extracted the most frequent terms in the domain and organized them into a hierarchical coding system—ROP Minimal Standard Terminology, which contains 62 core concepts in 4 categories. This terminology has been successfully used to provide highly structured and semantic-rich clinical data in several ROP-related applications.

## 1. Introduction

Retinopathy of prematurity (ROP) is a vaso-proliferative retinal disease affecting premature and low birth-weight infants. It is one of the main causes of children blindness worldwide. With the advancement of perinatal care quality, the survival rate of premature infants increases steadily, making ROP an unneglectable problem in both developed and developing countries. In China alone, there are about two million premature babies born annually. The incidence rate of ROP among premature babies is about 10% [1]. A conservative estimate of annual ROP infants is 200,000. The timely screening and intervention have become a huge problem worldwide.

To address this problem, four years ago, we initiated the CMS-R (Case Management System for ROP) project. This system is designed to support effective clinical data management and provide cross-regional telemedicine of ROP screening. One prerequisite of CMS-R is a well-defined domain-specific terminology. Such a terminology is essential for achieving SDE (structured data entry) and generating highly structured clinical data. It can also be used for future data exchange with external health information systems. This paper will introduce a ROP-specific terminology developed for CMS-R.

## 2. Related Work

Terminology, a.k.a. controlled vocabulary, is a collection of terms with explicitly defined meanings and unique codes in a specific domain. In the medical domain, there are hundreds of openly published terminologies. Readers may refer to https://www.nlm.nih.gov/research/umls/sourcereleasedocs/index.html for a list of medical terminologies. The following are some of the most widely used biomedical terminologies.

ICD (International Classification of Diseases [2]) organizes disease terms in a hierarchical style according to their semantic relations. It is widely used in EMRS (Electronic Medical Record System) and HIS (Hospital Information System) as diagnostic codes. LOINC (Logical Observation Identifiers Names and Codes) [3] is a terminology of tests, measurements, and observations, which is widely used in LIS (Laboratory Information System). CPT (Current Procedural Terminology) [4] is a medical code set for medical services, surgeries, and procedures. CPT terms are often used for billing items in HIS. RxNorm [5] is a drug terminology, which is widely used in CPOE (Computerized Physician Order Entry). MTHMST (Metathesaurus Minimal Standard Terminology Digestive Endoscopy) [6] is a domain-specific in terminology for the endoscopy specialty, authored by ESGE (European Society of Gastrointestinal Endoscopy). GO (Gene Ontology) [7] is a terminology for molecular function, biological process, and cellular component. HPO (Human Phenotype Ontology) [8] provides a well-defined set of terms that describe human phenotypic abnormalities. SNOMED-CT (Systematized Nomenclature of Medicine-Clinical Terms) [9] is a rather comprehensive medical terminology, which uses a formally defined medical ontology as the backbone for concepts and terms. UMLS (Unified Medical Language System) [10] metathesaurus is a project initiated by US National Library of Medicine, aiming at mapping concepts in existing terminologies into a comprehensive metathesaurus ontology. The current UMLS version has integrated more than 200 existing terminologies.

Most biomedical terminologies are focused on a specific domain or developed for a special purpose. When it comes to a specific domain, such specialized terminologies have more advantages than general-purposed ones: (1) Expressiveness: some fine-grained concepts in a specific domain may not be directly available in general-purposed terminologies. For example, “Type 1 ROP” is a special concept in the ROP domain and is difficult to find an off-the-shelf item in existing terminologies. (2) Efficiency: a specially tailored terminology can be more coherent and efficient in expressing certain domain concepts. In such cases, general-purposed medical terminologies may have to use complex postcoordinated expressions or combinations of multiple terms. (3) Reasoning and inference: specialized terminologies can use hierarchical coding systems to facilitate reasoning and semantic query. For example, H35.0 (background retinopathy and retinal vascular changes) and H35.1 (retinopathy of prematurity) in ICD-10 are sibling concepts under the common parent concept H35 (other retinal disorders).

Currently, there is no specially tailored terminology for ROP, which has hindered the effective application of ROP-related systems. In this manuscript, we will introduce a domain-specific terminology for ROP and demonstrate several used cases of ROP-related applications.

## 3. Terminology Development

### 3.1. Clinical Settings and Materials

This study is conducted in Shenzhen Eye Hospital (SEH), a 200-bedded class III specialized hospital in China. SEH has long been providing ROP screening services for peripheral partner hospitals, including Shenzhen People's Hospital, Peking University Shenzhen Hospital, University of Hong Kong-Shenzhen Hospital, Shenzhen Maternal and Child Health Hospital, Meizhou People's Hospital (Guangdong Province, China), and Puning People's Hospital (Fujian Province, China). With more than 10 years of experience, SEH has accumulated more than 20,000 ROP infants' clinical data. Based on these historical data, we made a term frequency analysis (detailed analysis data can be downloaded from http://ropd.brahma.top/Assets/TermFrequency.xls.) to identify most frequently used terms in the ROP domain.

From the analysis, a total of 37,070 valid text strings are extracted, which correspond to 752 distinct narrative terms. We then sort the terms by their frequencies in descending order, to determine which terms are used most often. As the distinctive term number is not huge (752), the ophthalmologists manually coordinated (e.g., multiple free-text narrations of a same concept) these terms and reorganized them into a hierarchical concept tree.

### 3.2. The ROP_MST Terminology

Based on the above analysis, we built a hierarchical terminology—ROP_MST (ROP Minimal Standard Terminology), which contains 62 ROP-related core concepts in 4 primary categories (i.e., diagnosis, treatment, examination, and laterality). Each concept has a unique code and multiple aliases (equivalent narratives in different languages). The encoding rule is similar to ICD, that is, the code of a subordinate concept is prefixed by its superior concept code. For example, intravitreal injection (T004) is a parent concept of Ranibizumab intravitreal injection (T004.M001). Such encoding rule facilitates concept-level information retrieval and semantic reasoning. Users may refer to Tables 1–5 for the terminology.

## 4. Applications

### 4.1. Structured Data Entry

**Figure 1:**
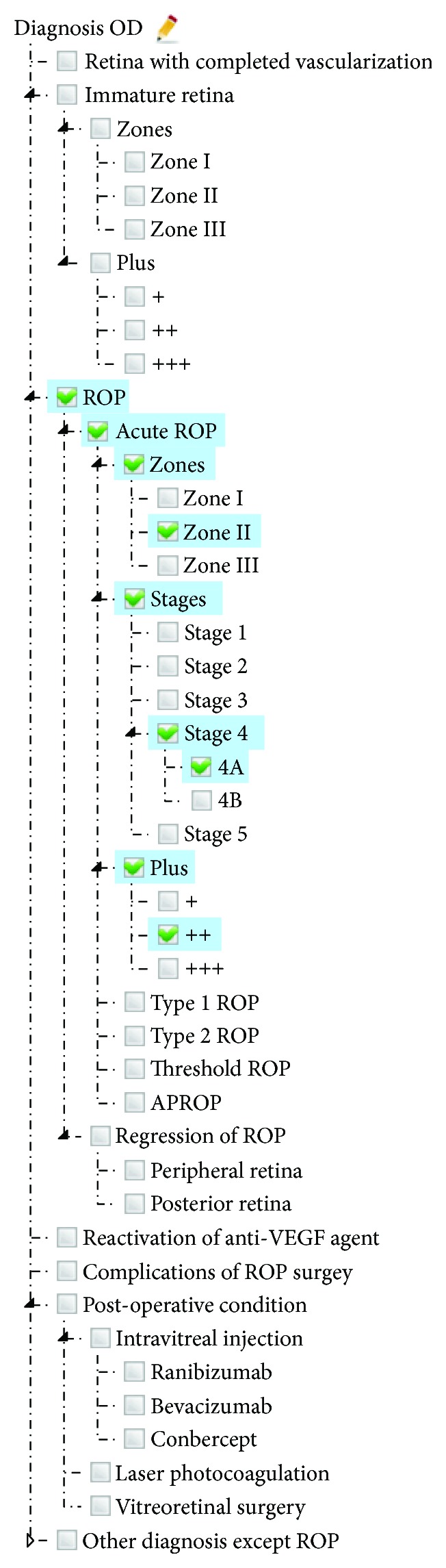
SDE (Structured Data Entry) for diagnosis in CMS-R.

A basic usage of ROP_MST is SDE, which ensures highly structured and semantic-rich clinical data for ROP-related information systems. In CMS-R (demo version: http://ropd.brahma.top), SDE is widely used. As shown in Figure 1, the diagnostic tree is arranged by terms' conceptual hierarchy. Users can click the triangle icon to expand or collapse branches. When user clicks a child node, all parent nodes along its path will also be selected. User can express complex conditions by selecting multiple nodes. For example, “ROP Zone II Stage 4A ++” can be expressed by *D002.A001*, *D002.A001.Z002*, *D002.A001.S004A*, and *D002.A001.P002*. When user saves patient data, the codes of the selected terms will be persisted in the server-side database. As each concept/term is explicitly assigned to a unique code, the potential ambiguity and chaos that arise from free-text input can be prevented.

### 4.2. Advanced Search

Information retrieval is a common task for clinical information systems, for example, searching qualified patients to be included in randomized clinical trials. As ROP_MST codes imply relations between subordinate and superior concepts, we can use it for advanced search. For instance, if the user wants to search all patients treated by intravitreal injection (T004), no matter the injection is Ranibizumab (T004.M001), Bevacizumab (T004.M002), or Conbercept, one simple search rule “[VisitTreatmentCode] == T004%” would suffice (“%” is a wild card and “T004%” means any code starting with “T004”). In contrast, the traditional way based on plain text matching usually requires users to enumerate all subordinate literal cases and write complex search patterns. Readers may access the advanced search function in CMS-R (http://ropd.brahma.top/search).

### 4.3. Reasoning to Get the Most Severe Diagnoses

Getting the most severe diagnosis based on multiple visit diagnoses is a very common task in ROP research. Traditionally, this job is done manually by physicians. With the help of ROP_MST, this can be automated by reasoning over the diagnosis codes. For instance, as ROP_MST defines fine-grained terms (i.e., zones, stages, and plus) for acute ROP (D002.A001), the severity of acute ROP can be judged by combining zone codes (D002.A001.Z001 > D002.A001.Z002 > D002.A001.Z003), stage codes (D002.A001.S001 < D002.A001.S002 < D002.A001.S003 < D002.A001.S004 < D002.A001.S005), and plus codes (D002.A001.P001 < D002.A001.P002 < D002.A001.P003). In CMS-R, an “induced most severe diagnosis” algorithm was designed to relieve users of manual data inputs.

### 4.4. Fundus Image Labeling Tool for Deep Learning

**Figure 2:**
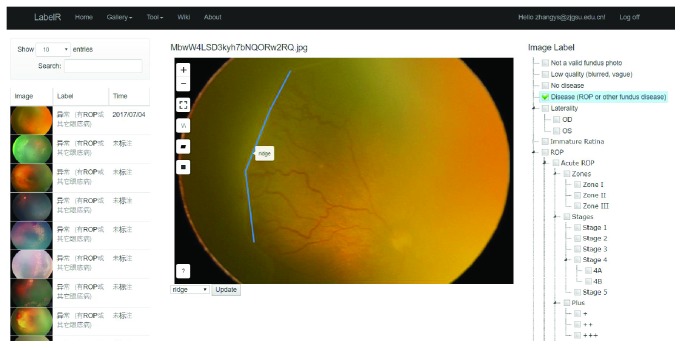
Fundus image labeling tool based on ROP_MST.

Computer-aided diagnosis based on fundus photography is a promising technology in ROP screening and telemedicine. Since the beginning of 2017, we have been using deep learning techniques to train a classifier to identify whether a fundus image has ROP or not. One prerequisite resource is a training set with high-quality class labels, and a “LabelR (Labeling Tool for ROP, http://label.brahma.top)” system was developed. LabelR allows user to assign multiple unambiguous and fine-grained diagnostic labels from ROP_MST to each fundus image (Figure 2).

## 5. Conclusions and Discussions

The first version of ROP_MST was designed in 2013 and has since then been evolving to better suit pediatric ophthalmologists' needs. Compared to other coding systems, the unique strength of ROP_MST is its specialty and domain orientation. All terms in ROP_MST are systematically organized by a hierarchical coding mechanism and are much easier for ROP-related applications. During research, we also encountered several issues that require concerns or future research.

### 5.1. Using Clustering Algorithms to Aggregate Terms

In building ROP_MST, the disambiguation of multiple literal strings for the same concept is performed manually by pediatric ophthalmologists. However, for other future ophthalmology terminologies, the total number of literal strings could be larger (say tens of thousands). For such cases, the manual operation would become unrealistic. A feasible solution would be designing a string similarity function (e.g., Levenshtein distance) and a text clustering algorithm (e.g., *k*-means).

### 5.2. Mapping with Existing Coding Systems

In order to integrate existing biomedical data encoded by traditional coding systems, it is essential to implement a terminology translation service. This service aims to map existing coding systems to ROP_MST, which could be a rather complicated task due to the heterogeneity between terminologies. Although several concepts can be directly mapped (e.g., “retinopathy of prematurity” (H35.1, ICD-10) ↔ “ROP” (D002) and “stage of retinopathy in retinopathy of prematurity” (422746009, SNOMED CT)↔ “ROP stage” (D002.A001.S)), others may involve the mapping of multiple-concept combinations between different terminologies.

## Figures and Tables

**Table 1:** Term categories.

Alias 1: English	Alias 2: Chinese	Alias 3: Japanese	Code	Coding system
Diagnosis	诊断	診断	D	ROP_MST
Treatment	治疗	治療	T	ROP_MST
Examination	检查	検査	E	ROP_MST
Laterality	眼别	目の左右差	L	ROP_MST

**Table 2:** Diagnostic terms.

Alias 1: English	Alias 2: Chinese	Alias 3: Japanese	Code	Coding system
Diagnosis	诊断	診断	D	ROP_MST
Retina with completed vascularization	视网膜完全血管化	網膜血管完全に成長した	D000	ROP_MST
Immature retina	视网膜发育不全	網膜血管形成不全	D001	ROP_MST
Zone	分区	ゾーン	D001.Z	ROP_MST
Zone I	1 区	ゾーン I	D001.Z001	ROP_MST
Zone II	2 区	ゾーン II	D001.Z002	ROP_MST
Zone III	3 区	ゾーン III	D001.Z003	ROP_MST
Plus disease	Plus 病变	プラス病変	D001.P	ROP_MST
+	+	+	D001.P001	ROP_MST
++	++	++	D001.P002	ROP_MST
+++	+++	+++	D001.P003	ROP_MST
ROP	早产儿视网膜病	未熟児網膜症	D002	ROP_MST
Acute ROP	急性 ROP	急性 ROP	D002.A001	ROP_MST
Zone	分区	ゾーン	D002.A001.Z	ROP_MST
Zone I	1 区	ゾーン I	D002.A001.Z001	ROP_MST
Zone II	2 区	ゾーン II	D002.A001.Z002	ROP_MST
Zone III	3 区	ゾーン III	D002.A001.Z003	ROP_MST
Stage	分期	ステージ	D002.A001.S	ROP_MST
Stage 1	1 期	ステージ 1	D002.A001.S001	ROP_MST
Stage 2	2 期	ステージ 2	D002.A001.S002	ROP_MST
Stage 3	3 期	ステージ 3	D002.A001.S003	ROP_MST
Stage 4	4 期	ステージ 4	D002.A001.S004	ROP_MST
Stage4A	4A 期	ステージ 4A	D002.A001.S004A	ROP_MST
Stage4B	4B 期	ステージ 4B	D002.A001.S004B	ROP_MST
Stage 5	5 期	ステージ 5	D002.A001.S005	ROP_MST
Plus disease	Plus 病变	プラス病変	D002.A001.P	ROP_MST
+	+	+	D002.A001.P001	ROP_MST
++	++	++	D002.A001.P002	ROP_MST
+++	+++	+++	D002.A001.P003	ROP_MST
Type 1 ROP	1 型 ROP	I 型未熟児網膜症	D002.A001.1	ROP_MST
Type 2 ROP	2 型 ROP	II 型未熟児網膜症	D002.A001.2	ROP_MST
Threshold ROP	阈值 ROP	閾値未熟児網膜症	D002.A001.3	ROP_MST
APROP	急进型后极部早产儿视网膜病	積極的な後方未熟児網膜症	D002.A001.4	ROP_MST
Regression of ROP	退行性 ROP	未熟児網膜変性症	D002.A002	ROP_MST
Posterior retina	后极	網膜後極部	D002.A002.Z001	ROP_MST
Peripheral retina	周边	網膜周辺部	D002.A002.Z003	ROP_MST
Reactivation of anti-VEGF agent	抗 VEGF 治疗后复发	抗 VEGF 剤の再活性化	D003	ROP_MST
Complications of ROP surgery	ROP 术后并发症	ROP 合併症	D004	ROP_MST
Postoperative condition	ROP 术后	ROP 手術の歴史	D005	ROP_MST
Vision	视力	視力	D00F.V	ROP_MST
Normal vision	正常视力	正常視力	D00F.V001	ROP_MST
Subnormal vision	低视力	異常視力	D00F.V002	ROP_MST
Blindness	失明	失明	D00F.V003	ROP_MST

APROP = aggressive posterior retinopathy of prematurity; VEGF = vascular endothelial growth factor.

**Table 3:** Treatment terms.

Alias 1: English	Alias 2: Chinese	Alias 3: Japanese	Code	Coding system
Treatment/management	治疗	治療	T	ROP_MST
Regular follow-up	定期复查	定期的なレビュー	T001	ROP_MST
Laser photocoagulation	激光光凝术	レーザー光凝固	T002	ROP_MST
Intravitreal injection	玻璃体腔注药术	ケナコルト硝子体内注入	T004	ROP_MST
Ranibizumab	雷珠单抗	ラニビズマブ	T004.M001	ROP_MST
Bevacizumab	贝伐单抗	ベバシズマブ	T004.M002	ROP_MST
Conbercept	康柏西普	コンバーセル	T004.M003	ROP_MST
Vitreoretinal surgery	玻璃体视网膜手术	網膜硝子体手術	T003	ROP_MST

**Table 4:** Examination terms.

Alias 1: English	Alias 2: Chinese	Alias 3: Japanese	Code	Coding system
Examination	检查	検査	E	ROP_MST
Metabolomics test	代谢组学检查	メタボロミクス テスト	E008	ROP_MST
Imaging	影像学检查	イメージング	E010	ROP_MST
Fundus photograph	眼底照相	眼底写真	E010.IM01	ROP_MST
FFA	眼底荧光素血管造影	眼底フルオレセイン造影	E010.IM02	ROP_MST
CT	CT	CT	E010.IM03	ROP_MST
MRI	核磁共振	MRI	E010.IM04	ROP_MST
Corneal topography	角膜地形图	角膜形状解析	E010.IM05	ROP_MST
OCT	OCT	OCT	E010.IM06	ROP_MST
US	超声	超音波検査	E010.IM08	ROP_MST
ERG	视网膜电图	網膜電記録	E015	ROP_MST
VEP	视觉诱发电位	視覚誘発電位	E016	ROP_MST

FFA = fundus fluorescein angiography; CT = computed tomography; MRI = magnetic resonance imaging; OCT = optical coherence tomography; US = ultrasonography; ERG = electroretinography; VEP = visual evoked potential.

**Table 5:** Laterality terms.

Alias 1: English	Alias 2: Chinese	Alias 3: Japanese	Code	Coding system
Laterality	眼别	目の左右差	L	ROP_MST
OD	右眼	右目	L001	ROP_MST
OS	左眼	左目	L002	ROP_MST
OU	双眼	両方の目	L003	ROP_MST

OD = oculus dexter (right eye); OS = oculus sinister (left eye); OU = oculus utro (both eyes).
